# Incretin-Based Therapies for Diabetic Complications: Basic Mechanisms and Clinical Evidence

**DOI:** 10.3390/ijms17081223

**Published:** 2016-07-29

**Authors:** Daiji Kawanami, Keiichiro Matoba, Kazunori Sango, Kazunori Utsunomiya

**Affiliations:** 1 Division of Diabetes, Metabolism and Endocrinology, Department of Internal Medicine, Jikei University School of Medicine, 3-25-8 Nishi-shinbashi, Minato-ku, Tokyo 105-8461, Japan; matoba@jikei.ac.jp (K.M.); kazu-utsunomiya@jikei.ac.jp (K.U.); 2 Diabetic Neuropathy Project, Department of Sensory and Motor Systems, Tokyo Metropolitan Institute of Medical Science, 2-1-6 Kamikitazawa, Setagaya-ku, Tokyo 156-8506, Japan; sango-kz@igakuken.or.jp

**Keywords:** incretin, DPP-4, glucose-dependent insulinotropic polypeptide (GIP), GLP-1, diabetes, diabetic complications, cardiovascular disease

## Abstract

An increase in the rates of morbidity and mortality associated with diabetic complications is a global concern. Glycemic control is important to prevent the development and progression of diabetic complications. Various classes of anti-diabetic agents are currently available, and their pleiotropic effects on diabetic complications have been investigated. Incretin-based therapies such as dipeptidyl peptidase (DPP)-4 inhibitors and glucagon-like peptide-1 receptor agonists (GLP-1RA) are now widely used in the treatment of patients with type 2 diabetes. A series of experimental studies showed that incretin-based therapies have beneficial effects on diabetic complications, independent of their glucose-lowering abilities, which are mediated by anti-inflammatory and anti-oxidative stress properties. Based on these findings, clinical studies to assess the effects of DPP-4 inhibitors and GLP-1RA on diabetic microvascular and macrovascular complications have been performed. Several but not all studies have provided evidence to support the beneficial effects of incretin-based therapies on diabetic complications in patients with type 2 diabetes. We herein discuss the experimental and clinical evidence of incretin-based therapy for diabetic complications.

## 1. Introduction

The number of patients with diabetes is increasing worldwide. Although remarkable advances have been made in developing novel agents against diabetes, therapies that directly target diabetic complications are still not available. Both experimental and clinical studies suggested that the inflammatory process plays an important role in the development of diabetic complications. Therefore, agents that can control not only hyperglycemia but also the inflammatory process may aid in the prevention and regression of diabetic complications. The potential beneficial effects of incretin-based therapies are increasingly recognized. DPP-4 inhibitors and GLP-1RA were originally developed to lower plasma glucose levels, but accumulating evidence shows that they have vascular protective effects as well, independent of their glucose-lowering abilities, in an incretin-dependent and incretin-independent manner. In this review article, we discuss our current understanding of incretin-based therapies against diabetic nephropathy, retinopathy, neuropathy, and macrovasculopathy.

## 2. Incretins as Therapeutic Targets of Diabetes

Incretins are gut-derived members of the glucagon superfamily that are released from the small intestine in response to nutrient ingestion. Glucagon-like peptide (GLP)-1 and glucose-dependent insulinotropic polypeptide (GIP) are major physiological incretins. They exert biological effects through their specific receptors: GLP-1 receptor (GLP-1R) and GIP receptor (GIPR), which are G-coupled protein receptors [1]. The binding of incretins to the receptors on pancreatic β cells leads to activation of adenylate cyclase-mediated signaling cascades. Accordingly, an increase in the intracellular cyclic adenosine monophosphate (cAMP)-mediated activation of protein kinase A stimulates the exocytosis of insulin-containing granules [1,2].

GLP-1 and GIP account for 50% to 70% of postprandial glucose-dependent insulin secretion [3]. These proteins have opposing effects on glucagon secretion from pancreatic α cells. GIP has been shown to stimulate glucagon secretion through GIPR in pancreatic α cells in a cAMP-dependent manner [1,4]. Enhanced glucagon secretion by GIP has been confirmed in patients with type 2 diabetes [4]. In contrast, GLP-1 has been shown to suppress glucagon secretion when plasma glucose levels are above the fasting level [5], meaning that GLP-1 does not suppress the counter-regulatory responses of glucagon against hypoglycemia. The inhibitory effects of GLP-1 on glucagon secretion are thought to be mediated by somatostatin. It has been shown that GLP-1 stimulates pancreatic somatostatin secretion [6], and blockade of somatostatin abolishes the inhibitory effect of GLP-1 on glucagon secretion [7]. Although the precise mechanism remains unknown, this evidence supports the notion of somatostatin-dependent glucagon inhibition by GLP-1.

GIP and GLP-1 have very short half-lives (approximately 1–2 min) because they are quickly degraded by DPP-4 (also known as CD26), which drastically reduces their activity [8]. DPP-4 is a widely expressed serine peptidase that exists in various cell types including vascular cells, renal cells, and T cells. DPP-4 inactivates peptides with an alanine, proline, or serine residue in the penultimate position from the *N*-terminus [9,10]. In addition to its membrane-bound form, DPP-4 also circulates as a soluble form in the plasma, which lacks the cytoplasmic and transmembrane domain with preserved catalytic activity [11]. DPP-4 functions as a binding protein and is highly accessible to peptide substrates circulating through the gut, liver, lung, and kidney [12,13].

At present, DPP-4 inhibitors and GLP-1RA are available as incretin-based therapies. GLP-1RA is resistant to DPP-4 degradation. DPP-4 inhibitors inhibit DPP-4-mediated GIP and GLP-1 inactivation, thereby elevating the GLP-1 and GIP levels, although the extent of elevation (picomolar) is small compared with pharmacological supplementation with GLP-1 analogs (nanomolar) [9].

Incretin dynamics are impaired in type 2 diabetes. Clinical studies have shown that the incretin effect in diabetic patients is reduced compared to that in healthy individuals, although it still remains unclear whether this is a cause or consequence of diabetes [14,15,16]. Furthermore, DPP-4 activity has been shown to be elevated in both type 1 and type 2 diabetic subjects [17,18,19]. GIP-based therapy for diabetes was abandoned because the insulinotropic effect of GIP is reduced and GIP-dependent postprandial glucagon production is increased in type 2 diabetes [20,21]. In contrast, the insulinotropic effect of GLP-1 has been shown to be preserved in type 2 diabetes [4,21,22].

## 3. Organ Protective Effects of Incretins

Hyperglycemia is known to activate metabolic pathways, including the diacylglycerol (DAG)-protein kinase C (PKC) pathway, advanced glycation end-products (AGE) pathway, polyol pathway, and hexosamine pathway [23]. Activation of these signaling pathways induces inflammation and oxidative stress, which has been implicated in the pathogenesis of diabetic complications. Incretin-based therapies in experimental studies have been shown to attenuate diabetic vascular complications by inhibiting metabolic pathways such as the PKC pathway [24] and AGE pathway [25,26].

Accumulating evidence show that GLP-1 induces anti-inflammatory effects by downregulating ROS production and NF-κB activation in vascular cells [27,28] and renal cells [26,29]. GLP-1 exerts these beneficial effects via GLP-1R. In addition to a GLP-1-dependent mechanism, DPP-4 inhibitors also exert organ protective effects in part through GLP-1-independent mechanisms. DPP-4 has exopeptidase activity that cleaves dipeptides from the amino terminus of polypeptides with a proline or alanine at the second position [30]. DPP-4 has multiple substrates other than GLP-1, including brain natriuretic peptide (BNP) [31], substance P [32], neuropeptide Y (NPY) [33], stromal-derived factor 1α (SDF-1α) [34], and high-mobility group protein B1 (HMGB1) [35]. These substrates have been implicated in regulating vascular function such as vascular tone regulation, inflammation, cell migration, and cell differentiation [36]. For example, SDF-1α is a chemokine that attracts stem cell such as hematopoietic stem cells (HSCs) and endothelial progenitor cells (EPCs) [11]. Interestingly, the DPP-4 inhibitor linagliptin has been shown to reduce infarct size after myocardial ischemia in rats by inhibiting SDF-1α degradation, thereby enhancing the recruitment of CXC chemokine receptor 4 (CXCR4), a specific receptor for SDF-1α-positive circulating progenitor cells [37]. Similar effects mediated by DPP-4’s substrates can be expected in other organs, particularly in the kidney, in which DPP-4 is expressed at the highest level per organ weight [12]. Furthermore, a study of the tissue distribution of linagliptin found that the accumulation was highest in the kidney [38]. These findings suggest that the kidney is the organ where DPP-4 interacts with DPP-4 inhibitor.

**Figure 1:**
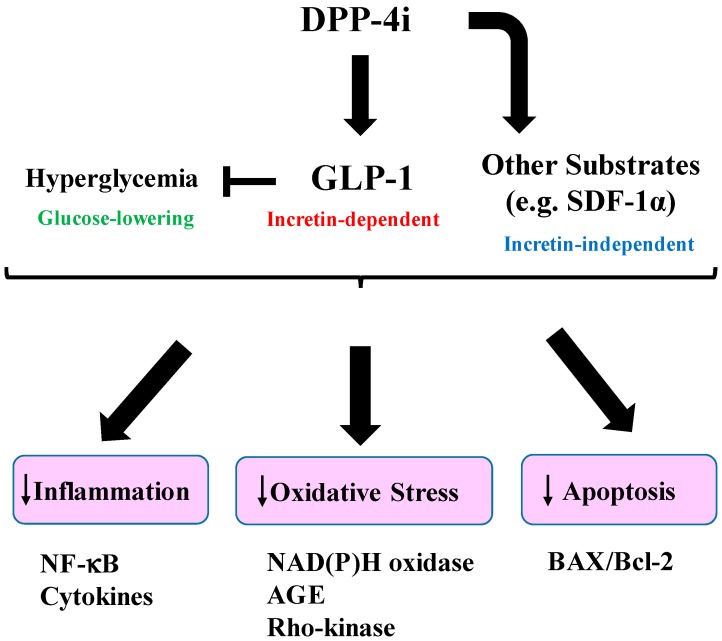
Mechanisms of beneficial effects of increased-based therapy. Dipeptidyl peptidase (DPP)-4 inhibition increases active glucagon-like peptide-1 (GLP-1) levels and GLP-1 signaling through its receptor. DPP-4 inhibition also inhibits degradation of its substrates other than GLP-1 (e.g., stromal-derived factor 1α (SDF-1α)), thereby activating incretin-independent signaling. GLP-1 inhibits inflammation and oxidative stress by downregulating inflammatory cytokine production (e.g., IL-1β, TNF-α), NF-κB, Rho-kinase activation, and the glycation end-products (AGE) pathway. GLP-1 inhibits apoptosis by decreasing the ratio of BAX/Bcl-2, which are a pro-apoptotic protein and an anti-apoptotic protein. The beneficial effects are also exerted via glucose-lowering by GLP-1.

**Figure 2:**
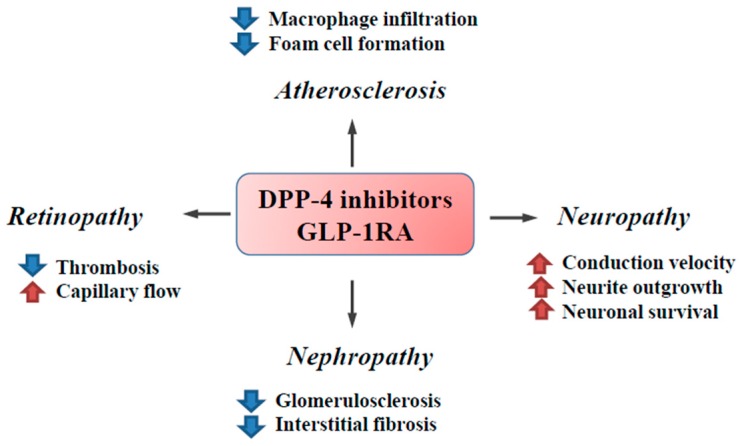
The effects of incretin-based therapies on diabetic complications. DPP-4 inhibitors and GLP-1RA attenuate diabetic complications through various beneficial effects. Incretin-based therapies have been shown to attenuate inflammation and oxidative stress, thereby inhibiting the fibrotic response in the kidney. In addition to these anti-inflammatory effects, endothelial dysfunction has been shown to be improved by incretin-based therapies, leading to improved capillary flow and inhibited thrombogenic activity in the retina. The anti-atherogenic effects of incretin-based therapies are mediated by the downregulation of inflammation, oxidative stress, and macrophage activation. The neuroprotective effects of incretin-based therapies are exerted by stimulating neurite growth and cell survival via the activation of GLP-1- and insulin-dependent signaling pathways. Blue arrows: decrease; Red arrows: increase.

Taken together, these findings suggest that the pleiotropic effects of incretin-based therapies are mediated by both incretin-dependent and incretin-independent mechanisms (Figure 1), which thereby exert beneficial effects on diabetic complications (Figure 2) (Table 1, Table 2 and Table 3).

## 4. Effects of Incretin-Based Therapies on Diabetic Nephropathy

### 4.1. Experimental Studies

Accumulating evidence shows the beneficial effect of incretin-based therapies on diabetic nephropathy. In rats, GLP-1R mRNA expression in the glomeruli and proximal tubules has been reported [70]. Furthermore, a study utilizing in situ hybridization showed that GLP-1R is expressed in the glomerular capillary wall and vascular structure but not in tubules in mice [71]. In humans, while one study found that GLP-1R was expressed in glomeruli and tubules [72], another study found it to be expressed dominantly in the proximal tubules [73]. To date, the localization of GLP-1R still remains controversial. These inconsistent results are thought to be due to the limited specificity and sensitivity of antibodies against GLP-1R [36].

It has been shown that renal DPP-4 expression and activity are upregulated in response to a high-fat diet in rats [74]. In addition, GLP-1R expression in the glomeruli has been shown to be downregulated in diabetic rats [75]. These observations indicate the potential usefulness of incretin-based therapies against diabetic nephropathy. A number of studies have investigated the effects of DPP-4 inhibitors in experimental diabetic models. Mega et al. revealed that the administration of sitagliptin attenuated glomerular, tubulointerstitial, and vascular lesions, accompanied by reduced lipid peroxidation in type 2 diabetic Zucker diabetic fatty (ZDF) rats [39]. Sitagliptin also has been shown to attenuate glomerulosclerosis and tubulointerstitial fibrosis by decreasing the levels of inflammatory cytokines such as IL-1β and TNF-α as well as cellular apoptosis in the kidney of ZDF rats [40]. However, it is difficult to determine the renoprotective effects beyond glucose reduction because DPP-4 inhibitors can improve glycemic control in type 2 diabetic models.

Interestingly, the administration of DPP-4 inhibitors has been shown to attenuate diabetic nephropathy in type 1 diabetic animal models independent of glucose-lowering. Kodera et al. reported that the DPP-4 inhibitor PKF275-055 attenuated urinary albumin excretion in STZ-diabetic rats, whereas glycemic control was not affected. A mechanistic analysis showed that PKF275-055 suppressed NF-κB activation, thereby inhibiting the expression of adhesion molecules and macrophage infiltration in the glomeruli [41]. The endothelial-mesenchymal transition (EndMT) plays an important role in the pathogenesis of diabetic kidney fibrosis. Kanasaki et al. showed that the DPP-4 inhibitor linagliptin ameliorates diabetic kidney fibrosis by EndMT, which is associated with the inhibition of DPP-4 protein expression by miR-29, the miR that negatively regulates 3′UTR of DPP-4 mRNA [42]. Of note, the attenuation of EndMT by linagliptin is thought to be drug-specific and not a class effect. Indeed, Shi et al. found that linagliptin but not sitagliptin inhibits TGF-β2-induced EndMT and DPP-4 3′UTR activity in human dermal microvascular endothelial cells [12]. The difference in the effects of these gliptins seems to be dependent on their ability to inhibit homo-dimer formation of DPP-4, which was observed only in linagliptin [12].

Mima et al. demonstrated that GLP-1R is downregulated by diabetes-induced PKCβ activation in glomerular endothelial cells. Mice overexpressing PKCβ2 in endothelial cells showed exaggerated albuminuria, and a mechanistic analysis revealed that PKCβ2 activation promotes ubiquitin-mediated GLP-1R degradation [76]. GLP-1RA has been shown to have direct renoprotective effects independent of the glucose-lowering ability in STZ-diabetic rats, a type 1 diabetic model [29,43]. The inhibition of oxidative stress and the inflammatory process are involved in the direct renoprotective effects of GLP-1RA. GLP-1-mediated PKA activation attenuates oxidative stress, as NAD(P)H oxidase is activated through PKA [77]. Furthermore, the GLP-1RA-mediated attenuation of albuminuria was associated with a reduction in the urinary 8-OHdG excretion, an oxidative stress marker [43]. It has been shown that GLP-1RA attenuates intercellular adhesion molecule (ICAM)-1 expression and macrophage infiltration in the kidney via the amelioration of oxidative stress and reduction of NF-κB expression [29]. Finally, GLP-1RA has been shown to inhibit AGE-mediated monocyte chemoattractant protein (MCP)-1 expression by inhibiting RAGE expression and subsequent ROS production in mesangial cells [26]. Consistent with these observations, GLP-1R-deficient C57/BL6 Akita mice showed increased albuminuria, mesangial expansion, and oxidative stress with decreased cAMP and PKA activity in the kidney [71]. Taken together, these findings show that GLP-1RA exerts a renoprotective effect independent of glucose-lowering by attenuating the inflammation induced by oxidative stress and NF-κB activation. A summary of incretin-based therapies on experimental models is shown in Table 1.

### 4.2. Clinical Studies

Clinical studies have shown that DPP-4 inhibitors attenuate albuminuria in type 2 diabetic subjects. Hattori et al. found that administration of sitagliptin (50 mg/day) for 6 months resulted in a significant reduction in urinary albumin excretion in 36 patients with type 2 diabetes whose HbA1c levels were higher than 6.5% [55]. In their study, sitagliptin improved glycemic control and lowered both systolic and diastolic blood pressures. Interestingly, significant reductions in highly sensitive C-reactive protein and soluble vascular cell adhesion molecule (VCAM)-1 were also observed [55], suggesting that the albuminuria reduction by sitagliptin was dependent on the glucose-lowering effect, as well as the blood pressure reduction and anti-inflammatory effects. Accordingly, Mori et al. investigated the effect of sitagliptin on albuminuria in comparison with other anti-diabetic agents [56]. Eighty-five patients (HbA1c < 6.5%) were allocated to either the sitagliptin group or the other anti-diabetic agents group. Improvement of glycemic control was observed in both groups, but a significant reduction in the urinary albumin excretion was obtained only in the sitagliptin group [56].

The Saxagliptin Assessment of Vascular Outcomes Recorded in Patients with Diabetes Mellitus-Thrombolysis in Myocardial Infarction (SAVOR-TIMI 53) trial showed improvement of albuminuria by saxagliptin in type 2 diabetic patients at risk for cardiovascular events [78]. The patients were stratified by the renal function as normal function (estimated glomerular filtration rate (eGFR) >50 mL/min/1.73 m^2^; *n* = 13,916), moderate renal impairment (eGFR 30–50 mL/min/1.73 m^2^; *n* = 2240), or severe renal impairment (eGFR <30 mL/min/1.73 m^2^; *n* = 336) and randomized to receive saxagliptin or placebo. After a two-year follow-up period, saxagliptin did not affect the risk of ischemic cardiovascular events, neither of which were affected by the renal function [78]. Importantly, saxagliptin reduced albuminuria, regardless of the baseline renal function. Given that the HbA1c reduction was significant in the saxagliptin group at two years (7.5% in saxagliptin vs. 7.8% in placebo, *p* < 0.01) [57], whether the saxagliptin-mediated reduction of albuminuria was due to a glucose-lowering or incretin-dependent mechanism remains unclear. Groop et al. showed a potential glucose-independent effect of linagliptin on albuminuria [58]. In their study, 217 type 2 diabetic patients with albuminuria under RAAS inhibitors were randomized to a placebo group or linagliptin group. Linagliptin treatment induced a significant reduction (32%) in the urinary albumin-creatinine ratio (ACR), and this finding was not associated with the magnitude of the control of the blood glucose and blood pressure [58].

Fujita et al. showed an important finding that supports the notion that DPP-4 inhibitors attenuate albuminuria beyond glucose-lowering [59]. They investigated the effect of the combination of DPP-4 inhibitors with ARB in type 2 diabetic patients with incipient nephropathy. The study consisted of three treatment periods: sitagliptin 50 mg/day for four weeks (first period), alogliptin 25 mg/day for four weeks (second period), and sitagliptin 50 mg/day for four weeks (third period) [59]. Intriguingly, switching from sitagliptin to alogliptin resulted in a decrease in the urinary levels of albumin and 8-hydroxy-2′-deoxyguanosine (8-OHdG), and an increase in the urinary cAMP levels and plasma levels of SDF-1α [59], suggesting that alogliptin can attenuate albuminuria by inhibiting oxidative stress through the reduction of the SDF-1α degradation by DPP-4. To further understand the potential renoprotective effects of linagliptin beyond its glucose-lowering abilities, the Efficacy, Safety & Modification of Albuminuria in Type 2 Diabetes Subjects with Renal Disease with LINAgliptin (MARLINA-T2D) study is currently in progress [79]. In this study, 350 inadequately controlled type 2 diabetic individuals with albuminuria were randomized to either the linagliptin group (5 mg/day) or placebo group in addition to receiving stable glucose-lowering background therapy for 24 weeks [79]. The results will provide novel evidence of the pleiotropic effects of linagliptin on diabetic nephropathy.

The beneficial effects of GLP-1RA on albuminuria in type 2 diabetic patients have also been reported. For instance, long-term treatment (one-year) of liraglutide has been shown to reduce albuminuria as well as lower the blood glucose and blood pressure [60,80]. The renoprotective effects of GLP-1RA appear to be exerted via the inhibition of the fibrotic process in the kidney. Zhang et al. demonstrated that exenatide can reduce urinary TGF-β1 and type IV collagen excretion in type 2 diabetic patients [61]. In their study, 31 type 2 diabetic patients with microalbuminuria were allocated to either the exenatide (initiated with 5 µg twice daily the first four weeks then increased to 10 µg twice daily) group or glimepiride (1–4 mg/day) group. All of the subjects were under metformin treatment (1.0–1.5 g/day). After 16 weeks, exenatide but not glimepiride treatment significantly reduced the urinary excretion of albumin, TGF-β1, and type IV collagen, with no significant difference in the glycemic control between the groups [61]. Taken together, these findings suggest that both DPP-4 inhibitors and GLP-1RA could be attractive therapeutic options against diabetic nephropathy. However, large randomized clinical trials are required to conclude the usefulness of incretin-based therapies for diabetic nephropathy. A summary of clinical studies that evaluate the effect of incretin-based therapies is shown in Table 2.

## 5. Effects of Incretin-Based Agents on Diabetic Retinopathy

### 5.1. Experimental Studies

Retinal endothelial cell dysfunction plays an important role in the development of diabetic retinopathy because it causes pericyte loss and increases vascular permeability and leukocyte adhesion, all of which are key features in diabetic retinopathy [44,81]. Blood-retinal barrier (BRB) breakdown is an early step of vascular permeability that can be induced by disruption of tight junctions (TJs) of endothelial cells [82].

DPP-4 inhibitors and GLP-1RA have been shown to exert beneficial effects on these changes. Increased DPP-4 activity in the retina has been reported in STZ-diabetic rats [44]. GLP-1R is reported to be expressed abundantly in the retina of humans and mice [47]. However, the regulation of GLP1-R under diabetic condition remains inconclusive. A study utilizing STZ-diabetic rats demonstrated that hyperglycemia downregulates GLP-1R expression in the retina [83], although Hernandez et al. found no significant differences in the GLP-1R expression levels in retinas derived from diabetic patients and db/db mice versus those from non-diabetic controls despite GLP-1 levels being lower in the retinas of those with diabetes [47]. Goncalves et al. demonstrated that sitagliptin inhibits BRB breakdown in both type 1 and type 2 diabetic models by preventing the changes in the endothelial subcellular distribution of the TJ proteins, inflammatory cytokines such as IL-1β, and cell death by apoptosis in diabetic retinas [44,45]. They also demonstrated that sitaglitpin prevented the diabetes-induced reduction in the adhesion ability of endothelial progenitor cells (EPCs) to the retinal vessels [44]. Similarly, vildagliptin has been shown to inhibit inflammation and thrombogenic reactions in the retina of Otsuka Long-Evans Tokushima Fatty rats (OLETF rats), a model of obese type 2 diabetes [46]. Both systemic and topical administration of GLP-1RA have been shown to inhibit retinal neurodegeneration such as glial activation and retinal apoptosis in db/db mice independently of the glucose-lowering effect [47]. From a mechanistic standpoint, GLP-1RA exerted these beneficial effects through a significant reduction of retinal glutamate and stimulation of prosurvival signaling pathways by increasing the pAKT/AKT ratio [47]. GLP-1RA has been reported to attenuate ischemia-reperfusion-induced BRB damage as well as inflammatory cytokine production by microglia activated by the inhibition of NF-κB activation [84].

### 5.2. Clinical Studies

Retinal hyperperfusion is an early hemodynamic change that occurs prior to clinical manifestations of diabetic retinopathy. A clinical trial to investigate the effect of saxagliptin on early retinal microvascular changes in patients with type 2 diabetes was performed [63]. In this study, 50 type 2 diabetic individuals without micro- or macro-vascular complications were randomized to the placebo group or saxagliptin (5 mg) group. After six weeks, the retinal arteriolar structure and retinal capillary flow (RCF) were assessed. Interestingly, administration of saxagliptin resulted in normalization of the RCF [63]. It has been shown that 10-month exenatide treatment resulted in transient worsening of diabetic retinopathy in 30% of diabetic subjects, which was associated with the rapid reduction in HbA1c levels [64,65]. However, a follow-up study revealed that sustained exenatide treatment improved or maintained stable diabetic retinopathy in 80% of patients who showed transient progression of diabetic retinopathy by exenatide [65]. This finding demonstrates the potential protective effect of GLP-1RA for diabetic retinopathy. However, given that significant improvement of glycemic control was obtained in both studies, it remains uncertain whether these observations are independent of or dependent on the glucose-lowering effect. Further study will be required to elucidate the direct beneficial effect of DPP-4 inhibitors on diabetic retinopathy.

## 6. Effects of Incretin-Based Agents on Diabetic Neuropathy

### 6.1. Experimental Studies

GLP1-R is expressed in the nervous tissues, including sensory neurons and Schwann cells in dorsal root ganglia (DRG). Sango & Utsunomiya demonstrated that GLP-1R is expressed predominantly in large and small peptidergic DRG neurons rather than small non-peptidergic neurons [85]. However, the expression of GLP-1RA is not altered under diabetic conditions.

GLP-1RA has been shown to exert beneficial effects on diabetic neuropathy in STZ-induced diabetic rats, independent of the glucose-lowering effect. Himeno et al. showed that four-week administration of GLP-1RA restored motor and sensory nerve conduction velocities (NCV) and hypoalgesia [50]. Furthermore, GLP-1RA has been shown to activate the ERK signaling pathway in peripheral neurons and/or Schwann cells derived from diabetic rats and mice, thereby protecting against the reduction of motor nerve conduction velocity (NCV) [51]. Tsukamoto et al. demonstrated that GLP-1RA restored the reduced neurite outgrowth and viability of adult rat DRG neurons caused by the absence of insulin in culture medium and suppressed the activity of RhoA, a small GTP-ase binding protein that is an inhibitory regulator for peripheral nerve regeneration, in PC12 cells. Furthermore, these effects were attenuated by the phosphatidylinositol-3′-phosphate kinase (PI3K) inhibitor LY294002, indicating that GLP-1RA enhances neurite outgrowth and neuronal survival through the activation of the PI3K signaling pathway, which negatively regulates RhoA activity [86].

DPP-4 inhibitors have been shown to inhibit diabetic neuropathy in both type 1 and type 2 diabetic rodent models. Jin et al. showed that vildagliptin protected STZ-induced diabetic rats from peripheral nerve degeneration by ameliorating decreases in the intraepidermal nerve fiber density [48]. Accordingly, Bianchi et al. investigated the protective and therapeutic effects of vildagliptin on diabetic neuropathy in STZ-induced diabetic rats. They observed that the vildagliptin analog PKF275-055 partially counteracted the reduction in the NCV but failed to improve the mechanical and thermal sensitivity of diabetic rats in prevention and protection experiments. However, they showed that PKF275-055 treatment restored mechanical sensitivity thresholds by 50% and progressively improved changes in the thermal responsiveness in therapeutic experiments [49]. Tsuboi et al. studied the effects of vildagliptin on diabetic neuropathy in more detail. They demonstrated that the administration of vildagliptin improved NCV in Goto-Kakizaki (GK) rats. Vildagliptin ameliorated delayed NCV and neuronal atrophy and reduced the expression of calcitonin-gene-related peptide (CGRP), a potent vasodilator of epineurial arterioles [87], as well as lowered the intraepidermal nerve fiber density in GK rats. Similarly, vildagliptin restored impaired NCV in STZ-induced diabetic mice [88]. From a mechanistic standpoint, vildagliptin corrected the impaired phosphorylation of cAMP response element binding protein (CREB), protein kinase B/Akt (PKB/Akt), S6-ribosomal protein (S6RP), and insulin receptor substrate (IRS) 2 in DRG of diabetic models, suggesting that vildagliptin restores the diabetes-induced impairment of GLP-1 and insulin signaling that play important roles in neurite growth and cell survival, thereby exerting protective effects against diabetic neuropathy [88]. Alogliptin has also been shown to improve the NCV in STZ-induced diabetic rats by improving CGRP-mediated vascular relaxation in epineurial arterioles [89].

### 6.2. Clinical Studies

Only one clinical study has investigated the effects of incretin-based therapy on diabetic neuropathy. Jaiswal et al. performed an open-label randomized study to evaluate the effects of GLP-1RA exenatide on diabetic peripheral neuropathy (DPN) as well as cardiovascular autonomic neuropathy (CAN) in subjects with type 2 diabetes [66]. In this study, 46 type 2 diabetic subjects with mild-moderate DPN were randomized to a twice daily exenatide group (*n* = 22) or daily insulin glargine (*n* = 24). After 18 months of follow-up, no significant differences were observed in the prevalence of confirmed clinical neuropathy, intra-epidermal nerve fiber density, and nerve conductions studies. Furthermore, there were no significant changes in the measures of CAN [66]. Glycemic control was similar in both groups [66]. Although GLP-1RA did not induce any marked beneficial effect in this study, further studies with different design will be required to conclude the clinical usefulness of incretin-based therapies.

## 7. Diabetic Macrovascular Complications

### 7.1. Experimental Studies

A series of experimental studies demonstrated the anti-atherosclerotic effects of incretin-based therapies in non-diabetic and diabetic animal models [90]. A study in STZ-induced diabetic apoE-deficient mice demonstrated that administration of alogliptin reduced the build-up of atherosclerotic plaque by inhibiting toll-like receptor 4-mediated IL-6 and IL-1β upregulation [52]. Consistently, vildagliptin has been shown to suppress atherosclerotic lesions by inhibiting macrophage accumulation and foam cell formation in STZ-induced apoE-null mice as well as db/db mice. Interestingly, these beneficial effects of DPP-4 inhibitors were mediated, at least in part, through GLP-1 and GIP, because both incretin receptor blockers induced partial but not complete attenuation of vildaglitpin’s anti-atherosclerotic effects [91]. Indeed, native incretins (both GLP-1 and GIP) have been shown to attenuate atherosclerotic lesions and macrophage infiltration in the aortic wall in apoE knockout mice [92,93]. Vildagliptin likely inhibited the activation of monocytes rather than macrophages because the expression of GIPR and GLP-1R were dramatically suppressed by differentiation of monocytes into macrophages [91]. Furthermore, DPP-4 inhibitors have been shown to attenuate atherosclerotic lesion in diabetic models such as ZDF rats [53] and high-fat diet low density lipoprotein (LDL)-receptor-deficient mice [94] via the downregulation of oxidative stress, chemokine production, and monocyte recruitment by inhibiting Rac activation [53,94].

Similarly, GLP-1RA has been shown to attenuate atherosclerosis in diabetic animal models. Arakawa et al. found that exendin-4 reduced monocyte adhesion to the endothelium of aorta, thereby leading to the suppression of atherosclerotic lesions in apoE knockout mice [95]. Tang et al. demonstrated that sitagliptin as well as exenatide administration improved endothelial dysfunction in STZ-induced diabetic rats [54]. From a mechanistic standpoint, these drugs recovered the diabetes-induced impairment of vasorelaxation; increased the serum NO levels and reductions of serum endothelin-1 and inflammatory cytokine levels such as VCAM-1, tumor necrosis factor (TNF)-α, and IL-6 levels; and inhibited the ROS production in the aorta [54], suggesting that incretin-based therapies improve diabetes-induced endothelial dysfunction by inhibiting inflammation and oxidative stress. Indeed, GLP-1RA has been shown to inhibit high-glucose mediated inductions of NAD(P)H oxidases such as p47^phox^ and gp91^phox^ [27]. The cAMP/PKA-mediated inhibition of small GTPase-binding protein Rho and its effector Rho-kinase, important factors in the pathogenesis of diabetic complications [23], are involved in the beneficial effects of GLP-1 on oxidative stress in endothelia cells [27] and the aorta [54] under diabetic conditions.

### 7.2. Clinical Studies

Three large randomized trials—SAVOR-TIMI 53 [57], Examination of Cardiovascular Outcomes with Alogliptin versus Standard of Care (EXAMINE) [67], and Trial Evaluating Cardiovascular Outcomes with Sitagliptin (TECOS) study [68]—did not show any significant reductions in the rates of cardiovascular events by DPP-4 inhibitors in patients with type 2 diabetes. In addition, a meta-analysis (total of 75 studies comprising 45,648 patients with type 2 diabetes) demonstrated no significant protective effect of incretin-based therapies against cardiovascular events [96]. An unexpected finding in SAVOR-TIMI53 was an increased incidence of heart failure hospitalization [57], but this observation was not observed in EXAMINE or TECOS.

Data that support the anti-atherosclerotic effect of incretin-based therapies are emerging. Recent studies have shown that aloglitpin and sitagliptin prevent the progression of carotid atherosclerosis in patients with diabetes [97,98]. Furthermore, a meta-analysis demonstrated that GLP-1-based therapy has beneficial effects on atherosclerotic markers (brain naturetic peptide, high-sensitivity C-reactive protein, plasminogen activator inhibitor-1, total cholesterol, LDL cholesterol, and triglycerides) in patients with type 2 diabetes [99]. The evaluation of lixisenatide in acute coronary syndrome (ELIXA) investigated the effect of GLP-1RA lixisenatide on cardiovascular morbidity and mortality in type 2 diabetic patients with recent acute coronary syndrome, but no significant differences in the rates of cardiovascular events were noted [69]. Recently, the Liraglutide Effect and Action in Diabetes: Evaluation of Cardiovascular Outcome Results (LEADER) trial provided evidence that liraglutide reduces the rates of cardiovascular events in patients with type 2 diabetes and high cardiovascular risk [62]. In this study, a total of 9340 type 2 diabetic patients who had HbA1c levels of ≥7.0% were assigned to either a liraglutide (1.8 mg) or placebo group [62]. The primary composite outcome in the time-to-event analysis was the first occurrence of cardiovascular death, nonfatal myocardial infarction, or nonfatal stroke. After a median follow-up of 3.8 years, the primary composite outcome was significantly lower in the liraglutide group than in the placebo group (HR, 0.87: 95% CI: 0.78–0.97; *p* < 0.001 for noninferiority; *p* = 0.01 for superiority). The administration of liraglutide significantly reduced the rates of death from cardiovascular causes (HR, 0.78: 95% CI: 0.66–0.93; *p* = 0.007) and death from any cause (HR, 0.85: 95% CI: 0.74–0.97; *p* = 0.02) [62]. Of note, the rate of incidence of a composite outcome of renal or retinal microvascular events (nephropathy (defined as the new onset of macroalbuminuria or a doubling of the serum creatinine level and an eGFR of ≤45 mL/min/1.73 m^2^, the need for renal replacement therapy, or death from renal disease) and retinopathy (defined as the need for retinal photocoagulation or treatment with intra-vitreal agents, vitreous hemorrhaging, or diabetes-related blindness)) was significantly lower in the liraglutide group than in the placebo group (HR, 0.84: 95% CI 0.73 to 0.97; *p* = 0.02) [62]. The LEADER trial demonstrated for the first time the usefulness of GLP-1RA liraglutide in the reduction of the rate of cardiovascular events in patients with type 2 diabetes. These beneficial effects of liraglutide may have been separate from the glucose-lowering effect, given the observation of significant mean differences in the change from baseline to 36 months of cardiovascular risk factors (weight loss (−2.3 kg), systolic blood pressure (−1.2 mmHg), and diastolic blood pressure (−0.6 mmHg)) by liraglutide compared to placebo [62]. Further investigations will be required to elucidate the mechanisms by which liraglutide provides cardiovascular benefit in patients with type 2 diabetes. A summary of the trials that investigated the effects of incretin-based therapies is shown in Table 3.

## 8. Conclusions

Incretin-based therapies are among the most important therapeutic options in diabetes and have revolutionized the treatment of diabetes. Both basic and clinical evidence show that incretin-based therapies have beneficial effects on diabetic complications. However, several points remain to be elucidated. From a basic mechanism perspective, the differential functions between GIP and GLP-1 in diabetic complications are unclear. DPP-4 inhibitors and GLP-1RA are similar in their pleiotropic effects, but they have different pharmacologic actions because the former increases both GIP and GLP-1 levels. Furthermore, DPP-4 inhibitors exert beneficial effects via the DPP-4 substrate. Clarifying the role of GIP in diabetic complications may address the utility of incretin-based drugs. In addition, further studies will need to examine whether or not a combination of DPP-4 inhibitors and GLP-1RA can exert more potent beneficial effects on diabetic complications than either drug alone. Addressing these issues will help researchers develop a novel therapeutic strategy against diabetic complications using incretin-based therapies.

## Figures and Tables

**Table 1:** Summary of the effects of incretin-based therapies on experimental models. The beneficial effects of DPP-4 inhibitors and GLP-1 RA on diabetic microvascular and macrovascular complications have been reported.

Complication	Model	Drug/Dose/Duration	Major Effects
Nephropathy	ZDF rats [39]	Sitagliptin,10 mg/kg, 6 weeks	↓Glomerular lesion
	ZDF rats [40]	Sitagliptin,10 mg/kg, 6 weeks	↓Glomerulosclerosis
			↓Tubulointerstitial fibrosis
	STZ-diabetic rats [41]	PKF275-055, 3 mg/kg, 8 weeks	↓Inflammation
	STZ-diabetic mice [42]	Linagliptin, 5 mg/kg, 4 weeks	↓Kidney fibrosis
	STZ-diabetic rats [29]	Exendin-4, 10 mg/kg, 8 weeks	↓Inflammation
	STZ-diabetic rats [43]	Liragltuide, 0.3 mg/kg, 8 weeks	↓Oxidative stress
Retinopathy	STZ-diabetic rats [44]	Sitagliptin, 5 mg/kg, 2 weeks	↓Blood-retinal barrier breakdown
			↓Inflammation
			↓Neuronal apoptosis
	ZDF-rats [45]	Sitaglitpin 10 mg/kg, 6 weeks	↓Inflammation
			↓Retinal cell apoptosis
	OLETF rats [46]	Vildagliptin 3 mg/kg, 10 weeks	↓Thrombogenic reactions
	db/db mice [47]	Liraglutide 400 μg/kg, 15 days	↓Retinal neurodegenartion
Neuropathy	STZ-diabetic rats [48]	Vildagliptin 0.3 or 10 mg/kg, 32 weeks	↓Peripheral nerve degeneration
	STZ-diabetic rats [49]	PKF275-055 3 mg/kg, 4 or 5 weeks	↑NCV
	STZ-diabetic mice [50]	Exendin-4 10 nmol/kg, 4 weeks	↑Neurite DRG outgrowth
			↑MNCV, SNCV
	STZ-diabetic mice [51]	Exenatide 0.3 pmoles/kg/min, 8 weeks (infusion)	↑MNCV
Macrovasculopathy	STZ-diabetic apoE-null mice [52]	Alogliptin 15 mg/kg, 24 weeks	↓Atherosclerotic plaque
	ZDF rats [53]	Sitaglitpin 10 mg/kg or Linaglitpin 3 mg/kg, 4 weeks	↑Vascular relaxation, ↓Oxidative stress
	STZ-diabetic rats [54]	Sitagliptin 30 mg/kg or	↓Inflammation
		Exenatide 30 μg/kg/12h (infusion), 12 weeks	↑Endothelial function

ZDF: zucker diabetes fatty; STZ: streptozotocin; OLETF: Otsuka Long-Evans Tokushima Fatty; MNCV: motor nerve conduction velocity; SNCV: sensory nerve conduction velocity; NCV: nerve conduction velocity; DRG: dorsal root ganglion; ↓: decrease; ↑: increase.

**Table 2:** Summary of clinical studies that evaluate the effect of incretin-based therapies on diabetic microvascular complications in patients with type 2 diabetes (T2D). The renoprotective effects of incretin-based therapies have been reported. Further investigations into the usefulness of incretin-based therapies on retinopathy and neuropathy should be performed.

Complication	Drug	Doses (Duration)	Patients	Endpoint
Nephropathy	Sitagliptin [55]	50 mg/day (6 months)	T2D patients (*n* = 36)	↓Albuminuria
	Sitagliptin [56]	50 mg/day (6 months)	T2D patients (*n* = 85)	↓Albuminuria
	Saxagliptin [57]	2.5 or 5 mg/day (2 years)	T2D patients (*n* = 16,492)	↓Albuminuria
	Linagliptin [58]	5 mg/day (6 months)	T2D patients (*n* = 217)	↓Albuminuria
	Alogliptin [59]	25 mg/day (4 weeks) (vs. Sitagliptin 50 mg/day) (cross over)	T2D patients (*n* = 12)	↓Albuminuria
	Liraglutide [60]	0.6-1.8 mg/day (1 year)	T2D patients (*n* = 84)	↓Albuminuria
	Exenatide [61]	10 μg twice daily (16 weeks) (5 μg twice daily (first 4 weeks)	T2D patients (*n* = 31)	↓Albuminuria
	Liraglutide [62]	1.8 mg/day (3.8 years)	T2D patients (*n* = 9340)	↓Composite outcome of renal and retinal microvascular events
Retinopathy	Saxagliptin [63]	5 mg/day (6 weeks)	T2D patients (*n* = 50)	Normalization of retinal capillary flow
	Exenatide [64]	N/A (300 days)	T2D patients (*n* = 165)	Transient worsening of diabetic retinopathy (DR)
	Exenatide [65]	N/A (430 days)	T2D patients (*n* = 39)	Improvement of DR
Neuropathy	Exenatide [66]	10 μg twice daily (18 months) (5 μg twice daily (first 4 weeks))	T2D patients (*n* = 46)	No changes in confirmed clinical neuropathy, cardiovascular autonomic neuropathy

N/A: Not available.

**Table 3:** Clinical trials that investigated the effects of incretin-based therapies on the cardiovascular outcome in patients with T2D. All of the studies shown here were performed with T2D patients at high risk of cardiovascular disease. To date, Liraglutide Effect and Action in Diabetes: Evaluation of Cardiovascular Outcome Results (LEADER) is the only study that showed superiority of incretin-based therapy against cardiovascular events compared to placebo.

Trial	Drug/Doses	Patients	Primary Composite Outcome	Result (Risk of Cardiovascular Events)
SAVOR-TIMI53 [57] (2.1 years)	Saxagliptin 2.5 mg or 5 mg/day (on the basis of estimated glomerular filtration rate (eGFR) at baseline)	T2D patients who had a history of, or were at risk for, cardiovascular events (*n* = 16,492)	Cardiovascular death, myocardial infarction, or ischemic stroke	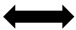 (no change)
EXAMINE [67] (1.5 years)	Alogliptin 6.25 mg or 12.5 mg or 25 mg (same as above)	T2D patients with either an acute myocardial infarction or unstable angina requiring hospitalization within the previous 15 to 90 days (*n* = 5380)	Cardiovascular death, nonfatal myocardial infarction, or nonfatal stroke	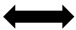
TECOS [68] (3.0 years)	Sitagliptin 50 mg or 100 mg/day (same as above)	T2D patients who had a history of major coronary artery disease, ischemic cerebrovascular disease, or atherosclerotic peripheral arterial disease (*n* = 14,671)	Cardiovascular death, nonfatal myocardial infarction, nonfatal stroke, or hospitalization for unstable angina	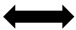
ELIXA [69] (2.1 years)	Lixisenatide 20 μg/day	T2D patients who had had a myocardial infarction or who had been hospitalized for unstable angina within the previous 180 days (*n* = 6068)	Cardiovascular death, myocardial infarction, stroke, or hospitalization for unstable angina	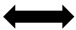
LEADER [62] (3.8 years)	Liraglutide 1.8 mg/day	T2D patients ≥50 years of age with at least one cardiovascular coexisting condition or ≥60 years of age with at least one cardiovascular risk factor (*n* = 9340)	Cardiovascular death, nonfatal myocardial infarction, or nonfatal stroke	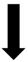 (decrease)
